# Intrahepatic Lymphangiogenesis Is Associated with Early Post-Hepatectomy Liver Regeneration, in Part via IL-6/STAT3 Signaling

**DOI:** 10.7150/ijms.106849

**Published:** 2026-01-14

**Authors:** Shudong Xie, Xiaofei Fan, Yang Liu, Hao Li, Chen Zhou, Chen Guo, Xiongzhuo Tang, Yingzi Ming, Pengpeng Zhang

**Affiliations:** 1 Transplantation Center, The Third Xiangya Hospital, Central South University, Changsha, Hunan, China.; 2 NHC Key Laboratory of Translational Research on Transplantation Medicine, Changsha, 410013, Hunan, China.; 3 Hunan Province Clinical Research Center for Infectious Diseases, Changsha, Hunan, China.; 4 Shandong Medical College, No.5460, Second Ring South Road, Jinan, Shandong 2500024, China.; 5 Animal Nutritional Genome and Germplasm Innovation Research Center, College of Animal Science and Technology, Hunan Agricultural University, Changsha, Hunan, China.

**Keywords:** lymphangiogenesis, lymphatic endothelial cells, liver regeneration, partial hepatectomy, VEGF-C, IL-6

## Abstract

**Background:** Insufficient liver regenerative capacity poses life-threatening risks to patients following partial hepatectomy (PHx), and existing clinical treatments provide limited options for enhancing regeneration. Lymphatic vasculature plays essential roles in the immune response through the uptake and transport of pathogens, antigens, inflammatory mediators, and antigen-presenting cells. Recent research has shown that lymphangiogenesis may contribute to both heart and bone regeneration. However, the role and underlying mechanisms of intrahepatic lymphangiogenesis in liver regeneration remain unclear.

**Methods:** Single-cell RNA sequencing was employed to identify dynamic changes in lymphatic endothelial cells (LyECs) in liver tissues following 70% PHx. A mouse model of liver regeneration was utilized to assess the contribution of intrahepatic lymphangiogenesis to the regenerative process after 70% PHx. Additionally, an adeno-associated virus overexpressing vascular endothelial growth factor-C (AAV-VEGF-C) was used to confirm the role of intrahepatic lymphangiogenesis in liver regeneration. qRT-PCR, western blotting and immunofluorescence staining were performed to investigate the potential underlying mechanisms. Furthermore, a neutralizing rat anti-murine anti-IL-6 antibody (anti-IL-6) was used to verify signaling pathway.

**Results:** Single-cell RNA sequencing analysis revealed dynamic changes of LyECs in liver tissues following 70% PHx. Consistent with these findings, the number and area of intrahepatic lymphatic vessels (LVs) around the portal tract significantly decreased on postoperative day 3 (POD3) in the mouse model of 70% PHx compared to the sham group, but the number and area recovered by POD7. Additionally, vascular endothelial growth factor-C(VEGF-C), a pro-lymphangiogenic growth factor, was found to increase in the liver of the 70% PHx mouse model. Stimulation of lymphangiogenesis with AAV-VEGF-C significantly accelerated liver regeneration and repair. Mechanistically, intrahepatic lymphangiogenesis might accelerate liver regeneration by the activation of the IL-6/STAT3 pathway. Blocking IL-6 reversed lymphangiogenesis-accelerated liver regeneration.

**Conclusions:** Intrahepatic lymphangiogenesis may contribute to early liver regeneration after PHx, with partial dependence on IL-6/STAT3 signaling. These findings support further investigation of lymphatic-modulating approaches as potential adjuncts to enhance postoperative recovery after PHx, particularly in selected contexts.

## Introduction

The liver has rapid and enormous regenerative capabilities following a significant loss of hepatic parenchymal cells after hepatic resection^1^. Liver function following partial hepatectomy (PHx) or living donor liver transplantation, especially in marginal living donor liver transplantation and patients with cirrhosis or acute on chronic liver failure, relies on the state of liver regeneration^2^, ^3^. Insufficient and delayed liver regeneration may result in serious adverse consequences, such as small-for-size syndrome (SFSS) and post-hepatectomy liver failure (PHLF)^4^, ^5^. SFSS may lead to a range of complications, including impaired liver function, graft dysfunction, delayed graft function, and increased risk of postoperative complications^6^. What's more, PHLF remains the most frequent cause in perioperative mortality after PHx, and there are no effective treatment options aside from liver transplantation, for which most patients do not qualify due to the shortage of liver donations^7^, ^8^. These mean that adequate and timely liver regeneration is a significant factor in determining a patient's recovery after liver resection. However, liver regeneration is a complex, highly orchestrated physiological process involving multiple cytokines, signaling pathways, and cell types. In addition, its mechanism is still unclear. Therefore, further studies are urgently required to identify novel therapeutic targets for patients with insufficient and delayed liver regeneration.

Lymphatic vessels (LVs) are a critical component of the vascular circulatory system, which plays essential roles in interstitial tissue fluid homeostasis. In addition, LVs also have important roles in the immune response through the uptake and transport of pathogens, antigens, inflammatory mediators, and antigen-presenting cells^9^. In the damaged tissue, an extensive lymphatic network contributes to normal tissue function in steady-state conditions and to tissue repair follow injury^10^. In the area adjacent to the regions of injury, the lymphatic vasculature undergoes extensive remodeling and expansion, including the formation of new LVs and enlargement of pre-existing vessels. Increasing evidence has demonstrated the contribution of lymphangiogenesis to tissue repair and regeneration^11^. In the heart, the lymphatic vascular system can influence cardiac repair and the regenerative potential of the myocardium^12^. Cardiac lymphangiogenic response significantly increased in myocardial infarction patients responding to ischemic injury^13^. Lymphangiogenesis is enhanced by ectopic VEGF-C stimulation following injury, leading to tissue repair and improvement in cardiac function. In diabetic wounds, reduced lymphangiogenesis was observed^14^. LVs in wounds contribute to the resolution of inflammation, tissue repair, and the overall restoration of tissue homeostasis by draining excess fluids and proteins, regulating tissue pressure, and facilitating immune responses^15^, ^16^. Promoting lymphangiogenesis accelerates diabetic wound healing^17^, ^18^. Furthermore, LyECs-derived interleukin-6(IL-6) can stimulate cell proliferation^19^, ^20^. When LyECs produce IL-6, it can act in an autocrine or paracrine manner, binding to its receptor (IL-6R) on the surface of LyECs or neighboring cells. This binding may activate the signal transducer and activator of transcription 3(STAT3) signaling pathway, which leads to the transcription of target genes involved in cell cycle regulation, survival, and proliferation. Therefore, lymphangiogenesis may influence the process of liver regeneration and repair.

Although lymphangiogenesis has been demonstrated to play a critical role in various tissue regeneration processes, including heart regeneration and bone regeneration^21^-^24^, its role in the process of liver regeneration have not been well determined^25^. Here our study showed a significant decrease in LVs numbers on POD3 in the process of liver regeneration in murine models following 70% PHx. In addition, enhancing lymphangiogenesis by using AAV-VEGF-C contributed to the advancement of liver regeneration and repair peak, whose mechanism might be associated with IL-6/STAT3 pathway. Therefore, lymphangiogenesis might serve as a potential strategy to promote liver regeneration and suggested its possible application in other reversible liver injuries.

## Materials and Methods

### Data source and preprocessing

Our study consisted of four groups, including mice following POD1, POD2, POD4 and Sham, which were obtained from the publicly available Gene Expression Omnibus (GEO) database including four normal liver tissue samples (https://www.ncbi.nlm.nih.gov/geo, GSE151309)^26^. The RNA sequencing data were analyzed such as for cell type identification and clustering analysis, with the Seurat program (http://satijalab.org/seurat/, R package, v5.0.3). Unique molecular identifier (UMI) count tables were loaded into R (R version 4.3.1) using the Read10X function. The criterion for filtering low-quality cells was nFeature RNA <500 or <800 or >3000, with a mitochondrial gene percentage of >30%. After filtering, 15,949 cells were retained for the downstream analyses, with 4891 cells from POD0 group, 3800 cells from POD1 group, 2775 cells from POD2 group and 4483 cells POD4. We identified 2000 highly variable genes using the “FindVariableFeatures” function and integrated the data using the “Harmony” R package. We used functions from Seurat v5.1.0 for dimension-reduction and clustering.

### Animals

The animal protocol was designed to minimize pain or discomfort to the animals. All animal experiments were performed according to the Institutional Animal Care and Use Committee guidelines of Central South University, which are in accordance with the National Institutes of Health guidelines. Male C57BL/6 mice (6 to 8 weeks old) were purchased from Hunan Slack Jingda Experimental Animal Company. Mice were housed in a specific-pathogen-free environment under controlled temperature (22 ± 2°C) and maintained on a 12h light/dark cycle. Mice had free access to regular food and autoclaved water before and after the surgery. The adeno-associated virus 8 (AAV) overexpressing VEGF-C (AAV-VEGF-C) used in our study is of mouse origin (Species: Mus musculus, GENE_ID: 22341), to match the murine model and ensure species compatibility. The viral vector used was GV388, which contains the following elements: CMV promoter, bGlobin intron, MCS-EGFP-3FLAG, WPRE, and hGH polyA signal. This AAV-based vector was obtained from GeneChem (Shanghai, China). In addition, the expression of AAV-VEGF-C was confirmed by qRT-PCR. For AAV-VEGF-C group, mice (male, 6-8 weeks old) were treated with AAV-VEGF-C (1×10^11^viral genomes/mouse) and AAV-Vehicle group were treated with AAV (1×10^11^viral genomes/mouse) without VEGF-C via orbital intravenous injection one month before PHx. For anti-IL-6 group, mice were treated with AAV-VEGF-C for one month before PHx, and 2.0 mg/kg of a neutralizing rat anti-murine anti-IL-6 antibody (Cat. No. 554398, clone MP5-20F3, BD Biosciences, Heidelberg, Germany) was applied at 30 min and 48 h following 70% PHx^27^, which blocks both IL-6 classic and trans-signaling^28^. Meanwhile, Vehicle group was treated with rat IgG1 isotype control antibody (BD Biosciences) at the same time points.

### 70% PHx model

According to previous reports, a 70% PHx was performed^29^. Briefly, mice (6-8weeks old) were anesthetized by pentobarbital (0.3%, 30 mg/kg) intraperitoneally. After disinfection, mice underwent midline laparotomy. And then, the left lateral lobe and median lobe (70% of the liver) were ligated at the base using 4-0 silk and cut separately. The abdominal wall and the skin were sutured separately with a 4-0 silk suture. During the surgery, mice were placed on a warming pad. Additionally, the mice were placed in an incubator(37°C) for recovery after surgery. The mortality rate was less than 5%. At Sham, POD3 and POD7, mice were sacrificed and liver samples were collected. Blood was collected through inferior vena cava puncture and allowed to coagulate in room temperature for 30 min, then centrifuged in 1,000 g in 4°C for 5 min to retrieve blood serum. The liver weight to body weight percentage was measured, and liver tissues were snap-frozen in liquid nitrogen and stored at -80°C for further analysis.

### Regeneration liver calculation

The lobes were weighed, and the liver weight to body weight percentage was calculated (liver weight to body weight percentage = liver weight/body weight ×100%, g/g)^30^, ^31^.

### Culture of Human LyECs and VEGF-C Treatment

Primary human lymphatic endothelial cells (LyECs) were obtained from Heifei SynthBiological Engineering Company and cultured with Endothelial Cell Growth Medium (Cat#CM-H026Y, Procell, Wuhan, China) at 37 °C in an atmosphere of 5% CO2 in a humidified incubator. A suspension of human LyECs was loaded onto a 12-well plate (1×10^5^ cells/well) and VEGF-C (0, 10, 50 ng/mL, Cat# HY-P74474, MedChemExpress) with additional different concentrations was added to medium for 24h.

### Measurement of serum alanine aminotransferase (ALT) and aspartate transaminase (AST)

Serum ALT and AST activities were measured as indicators of hepatic injury and conducted as described in the manufacturer's protocols (C009-2-1 and C010-2-1, Nanjing Jiancheng Institute of Biotechnology, Nanjing, China).

### HE staining

Mice liver samples were fixed in 10% neutralized buffered formaldehyde at 4 ºC for 48 hours. Paraffin blocks were made and sections cut at a thickness of 4 μm. Paraffin sections were deparaffinized with xylenes and rehydrated by washing through a graded alcohol series to deionized water. The sections were stained by hematoxylin for 2 mins and eosin for 30s, and washed by warm water for 5mins. Then the sections were dehydrated by washing through a graded alcohol series to xylenes and mounted with cytoseal (Cat#8310, Thermo scientific, USA).

### Immunofluorescence staining

Paraffin sections were deparaffinized with xylenes and rehydrated by washing through a graded alcohol series to deionized water. The hydrated tissue sections were washed with phosphate-buffered saline (PBS). To retrieve antigens, the sections were incubated with 20 mM EDTA antigen retrieval solution (pH 9.0) for 20 min at approximately 100ºC. For staining, a Tyramide SuperBoostTM kit with Alexa FluorTM 488/555 Tyramide (Cat #B40932, Cat #B40933, Invitrogen) was used as follows. Peroxydase activity was blocked using Blocking Buffer for 60 min at room temperature. Then the sections were incubated with primary antibodies dissolved in 5% donkey serum solution containing 0.3% Triton at 4℃ overnight (anti-Ki67, Cat#HA721115, HUABIO,1:200; anti-Lyve-1, Cat#ab14917, Abcam,1:200). After washing three times with PBS, incubation with the poly-HRP-conjugated secondary antibody was performed for 60 min. Sections were then washed three times with PBS and incubated with Alexa FluorTM 488/555 tyramide reagent solution. After about 5 min, the reaction was stopped with Reaction Stop Reagent and slides were washed with PBS. For immunofluorescence co-staining of LYVE-1 and podoplanin (PDPN), after completion of LYVE-1 staining the sections were immersed in diluted citrate buffer (pH 6.0) and heated in a microwave oven at 100% power until boiling (approximately 1-2.5 min) for antigen retrieval. The sections were then incubated overnight at 4 °C with the primary anti-PDPN antibody (Cat# 14-5381-81, Invitrogen, 1:200) diluted in 5% donkey serum containing 0.3% Triton X-100. After three washes with PBS, the sections were incubated with a poly-HRP-conjugated secondary antibody (Alexa Fluor® 488, Cat#ab180063, Abcam,1:200) for 60 min. Finally, the cells were incubated with mounting medium with 4',6-diamidino-2-phenylindole (DAPI, Cat#S2110, Solarbio, Beijing, China) at room temperature.

### Acquisition and quantification of images

Images were documented using a DMI 3000B system (Leica Microsystems, Wetzlar, Germany). For quantification of Ki67 and Lyve-1 in portal tracts, ≥10 images (X100 magnification) were obtained per slide. The number of Ki67-positive cells and the number and area of LVs were measured by Image J (National Institutes of Health, Bethesda, MD, USA). LV area was assessed with the area of LVs / portal vein (PV) area. The ratio of each parameter was calculated and subjected to statistical analysis.

### Quantitative real-time polymerase chain reaction (qRT-PCR)

In accordance with manufacturer's instructions, total RNAs were extracted from liver samples or human LyECs treated with VEGF-C (Cat#AG21023, Accurate Biology, China). Subsequently, cDNA was synthesized using the obtained RNAs and an Evo M-MLV RT Kit (Cat#AG11728, Accurate Biology, China). Genetic expression level was quantified using a Roche LightCycler 480Ⅱ with SYBR Green Master Mix (Cat#RK21203, Abclonal Technology, China), and the expression level were calculated using the 2^-ΔΔCt^ method. GAPDH served as an internal reference for normalization. All primers used for qRT-PCR were synthesized by Sangon Biotech (Sangon, Shanghai, China). The primer sequences used are listed in Supplementary Table 1.

### Western blot

Liver tissues were placed in radioimmunoprecipitation assay (RIPA) buffer (MA0151, meilunbio, Wuhan, China) supplemented with protease inhibitor (Roche), phosphatase inhibitor (Roche) and 1 mM phenylmethylsulfonyl fluoride (PMSF) using MagNA Lyser homogenizer (Servicebio) to grind the lysed tissue. The above operations were performed on ice or 4℃. Proteins were separated by sodium dodecyl sulfate-polyacrylamide gel electrophoresis (SDS-PAGE), and transferred to polyvinylidene fluoride (PVDF) membranes (Millipore, CA, USA). After blocking with 5% BSA for 1 h at room temperature, a protein-loaded membrane was incubated with primary antibody overnight at 4°C and secondary antibody for 1 h. The signals were visualized with an enhanced chemiluminescence (ECL) kit (Biosharp, Anhui, China), photographed and measured with the VisionWorks system (Analytik Jena AG). Blots were then stripped with Restore PLUS Stripping buffer (Cat#46430, Thermo Scientific) to detect multiple target protein by using different antibodies. These results are representative of at least three independent experiments. The following antibodies were used: anti-GAPDH (1:5000, Cat#380626, ZEN BIO, China), anti-p-STAT3 (1:1000, Cat#381552, ZEN BIO, China), anti- STAT3 (1:1000, Cat#380907, ZEN BIO, China).

### Statistical analysis

Statistical analyses were performed using GraphPad prism 8.0 (GraphPad Software, CA) and the results were expressed as the mean ± standard error of mean (SEM). Comparison between two groups was carried out using the unpaired Student's t-test. Comparison between multiple groups was undertaken using one-way ANOVA. Differences were considered significant when *p* < 0.05 (∗∗∗*p* < 0.001, ∗∗*p* < 0.01, ∗*p* < 0.05, ns > 0.05).

## Results

### Single cell RNA-sequencing analysis revealed dynamic changes of LyECs within liver tissues following 70% PHx

**Figure 1:**
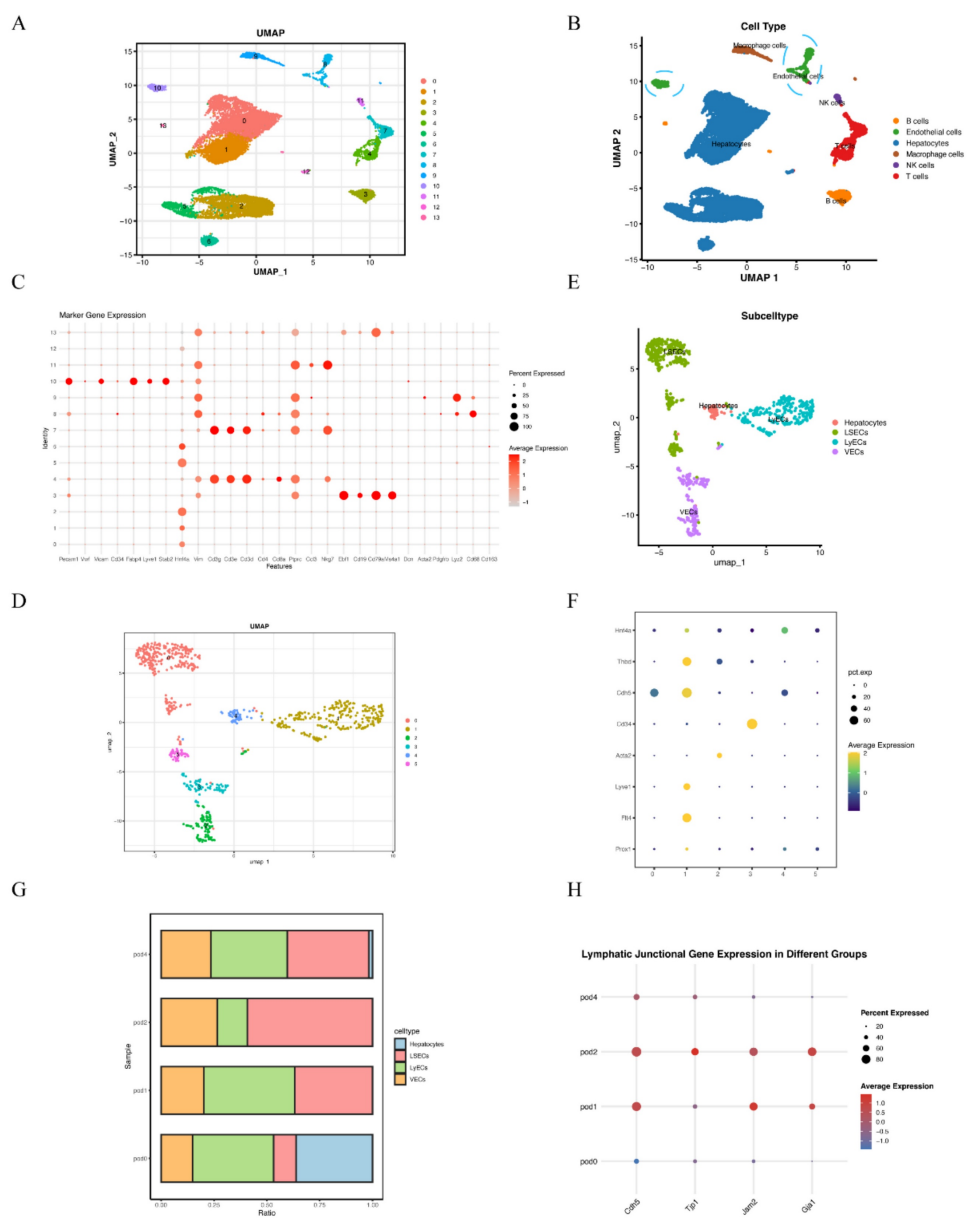
** Single cell RNA-sequencing revealed dynamic changes of LyECs within the process of liver regeneration following 70% PHx.** A: 15,949 cells from liver tissues of the POD0, POD1, POD2, and POD4 groups were annotated into 14 clusters and shown on UMAP plotting. B: A total of 14 different cell types were identified. C. The dotplot depicts classical marker genes of different cell types. D-E: 809 cells from endothelial cells of the POD0, POD1, POD2, and POD4 groups were re-annotated into 4 clusters and shown on UMAP plotting. F: The dotplot depicts classical marker genes of different endothelial cell types. G: The percentage change tendency of each LyECs cluster in the POD0, POD1, POD2, and POD4 groups. H. lymphatic junctional markers behave during PHx, such as such as Cadherin 5(Cdh5), Tight Junction Protein 1(Tjp1), Junctional Adhesion Molecule 2(Jam2), and Gap Junction Alpha-1(Gja1).

To characterize the role of lymphatic endothelial cells (LyECs) in liver tissues following 70% PHx, scRNA-seq was performed on liver tissue samples from the POD0, POD1, POD2, and POD4 groups. Quality control for each sample was done by assessing viability, RNA count, UMI count and mitochondrial gene ratio. After removing the suspicious double and low-activity cells, 4891 cells from the POD0 group, 3800 cells from the POD1 group, 2775 cells from the POD2 group and 4483 cells from the POD4 group were obtained for further analysis. The cells were re-clustered and annotated as Hepatocytes (Hnf4a^+^, Vim^-^), B cells (Ebf1^+^, Cd19^+^, Cd79a^+^, Ms4a1^+^), Endothelial Cells (Pecam1^+^, Vwf^+^, Mcam^+^, Cd34^+^, Fabp4^+^, Lyve1^+^, Stab2^+^), Macrophage cells (Lyz2^+^, Cd68^+^, Cd163^+^), NK cells (Ccl3^+^, Nkg7^+^) and T cells (Cd3g^+^, Cd3e^+^, Cd3d^+^, Cd4^+^, Cd8a^+^, Ptprc^+^) (Figure 1A and 1B). The classical marker genes of each subpopulation were shown in Figure 1C, and the TOP10 genes of these subpopulations were presented as in a heatmap (Supplementary material Figure S1). Due LyECs belong to endothelial cells, the endothelial cells subpopulations were further analyzed using scRNA-seq. A total of 809 Endothelial Cells from the POD0, POD1, POD2, and POD4 groups were classified into 6 clusters and annotated as Hepatocytes (Prox1^+^, Hnf4a^+^), LyECs (Prox1^+^, Flt4^+^, Lyve1^+^), LSECs (Prox1^+^, Flt4^low^, Lyve1^low^), and VECs (Acta2^+^, Cd34^+^) (Figure 1D and 1E). The classical marker genes were shown in Figure 1F and the TOP10 genes of endothelial cells were presented as in a heatmap (Supplementary material Figure S2). The ratio of LyECs was also assessed in different groups (Figure 1G). The ratio of LyECs presentated a decline in POD2 group compared to POD0, POD1 and POD4 groups. In additional, lymphatic junctional markers, such as Cadherin 5(Cdh5), Tight Junction Protein 1(Tjp1), Junctional Adhesion Molecule 2(Jam2), and Gap Junction Alpha-1(Gja1), exhibited a significant upregulation at POD1 and POD2, indicating enhanced lymphatic endothelial junctional activity during the early proliferative phase of liver regeneration. Those results suggested that the number and function of LyECs changed dynamically and LVs might play an important role in liver regeneration following 70% PHx.

### LVs decreased significantly on POD3 following 70% PHx

**Figure 2:**
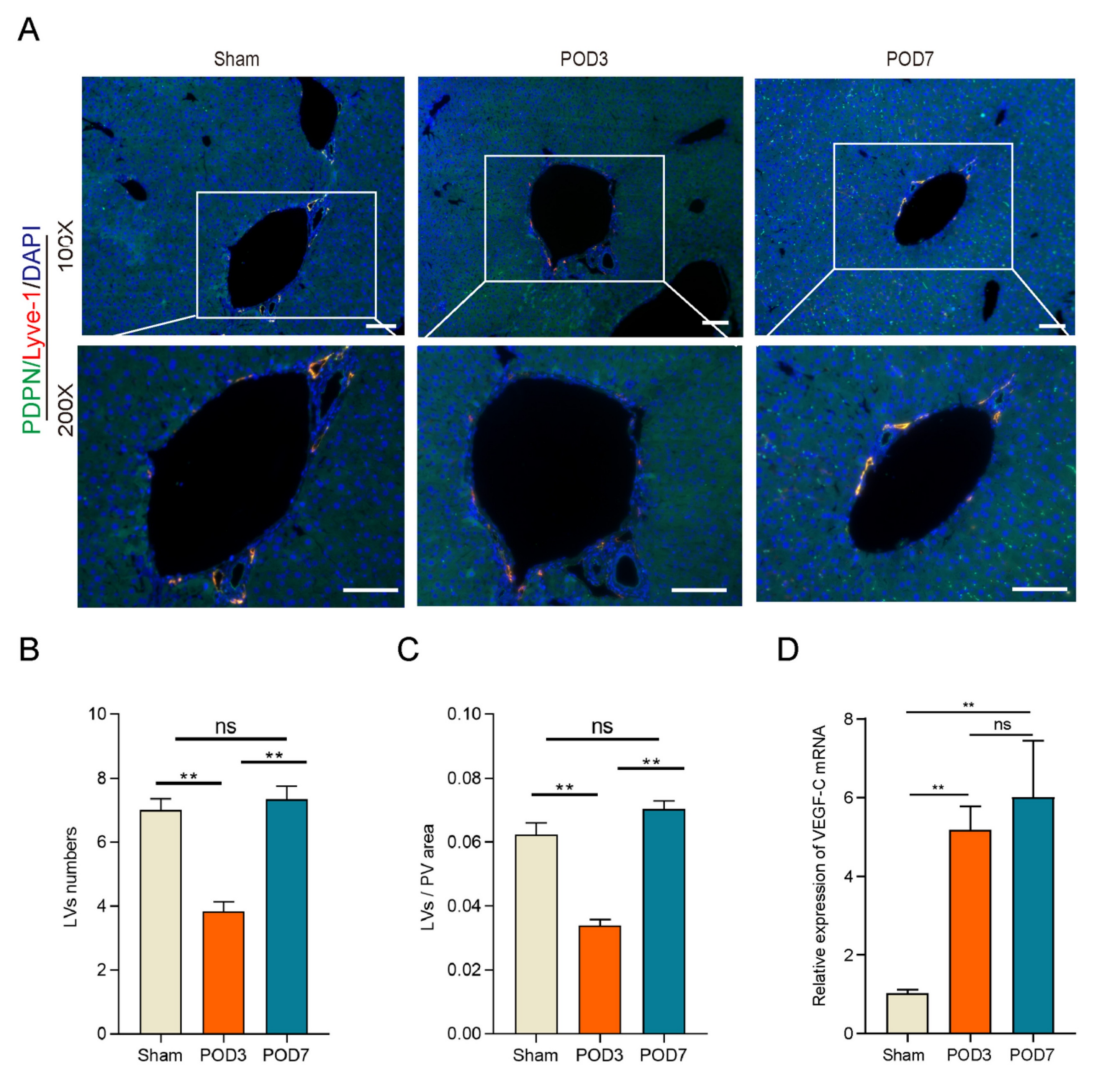
** Lymphangiogenesis decreased significantly on POD3 in the process of liver regeneration following 70% PHx.** A: Representatives of immunofluorescence co-staining of LYVE-1(red) and PDPN (green) in the liver on Sham, POD3, and POD7 groups (n = 5 per group, 5 biological replicates from 5 individual animals; original magnification 100X and 200X, scale bar: 100μm). B: Statistical analysis of LVs numbers in immunofluorescence staining. C: Statistical analysis of LVs area in immunofluorescence staining. D: The expression level of VEGF-C in the liver tissues was detected on Sham, POD3, POD7 groups via qRT-PCR (n=5 per group, 5 biological replicates from 5 individual animals). These results were obtained from at least three independent experiments. Values are presented as mean ± SEM. ∗∗*p* < 0.01, ∗*p* < 0.05, ns > 0.05.

To assess whether LVs play a role in liver regeneration following a 70% PHx, we examined LVs in the Sham, POD3, and POD7 groups. This approach provides a comprehensive view of both the early and later stages of regeneration, capturing the complete spectrum of regenerative processes and informing potential interventions^32^. Intrahepatic lymphatic vessels in the portal vein region were identified by immunofluorescence co-staining for lymphatic vessel endothelial hyaluronan receptor-1 (LYVE-1) and podoplanin (PDPN), two widely used markers of lymphatic endothelial cells (Figure 2A). As shown in the Figure 2B and 2C, compared to the Sham group and POD7 group, both the number and area of LVs decreased significantly on POD3. In addition, the number and area of LVs restored on POD7 without difference to the Sham group. Interestingly, the mRNA expression level of VEGF-C increased significantly in the process of liver regeneration (Figure 2D), which have been recognized playing a crucial role on lymphangiogenesis, the formation of new LVs from pre-existing ones. Increased VEGF-C expression may indeed act as a compensatory response to intrahepatic LVs damage^33^. All these data indicated that lymphangiogenesis might play a significant role in the process of liver regeneration.

### Administration of AAV-VEGF-C stimulates lymphangiogenesis in the liver

**Figure 3:**
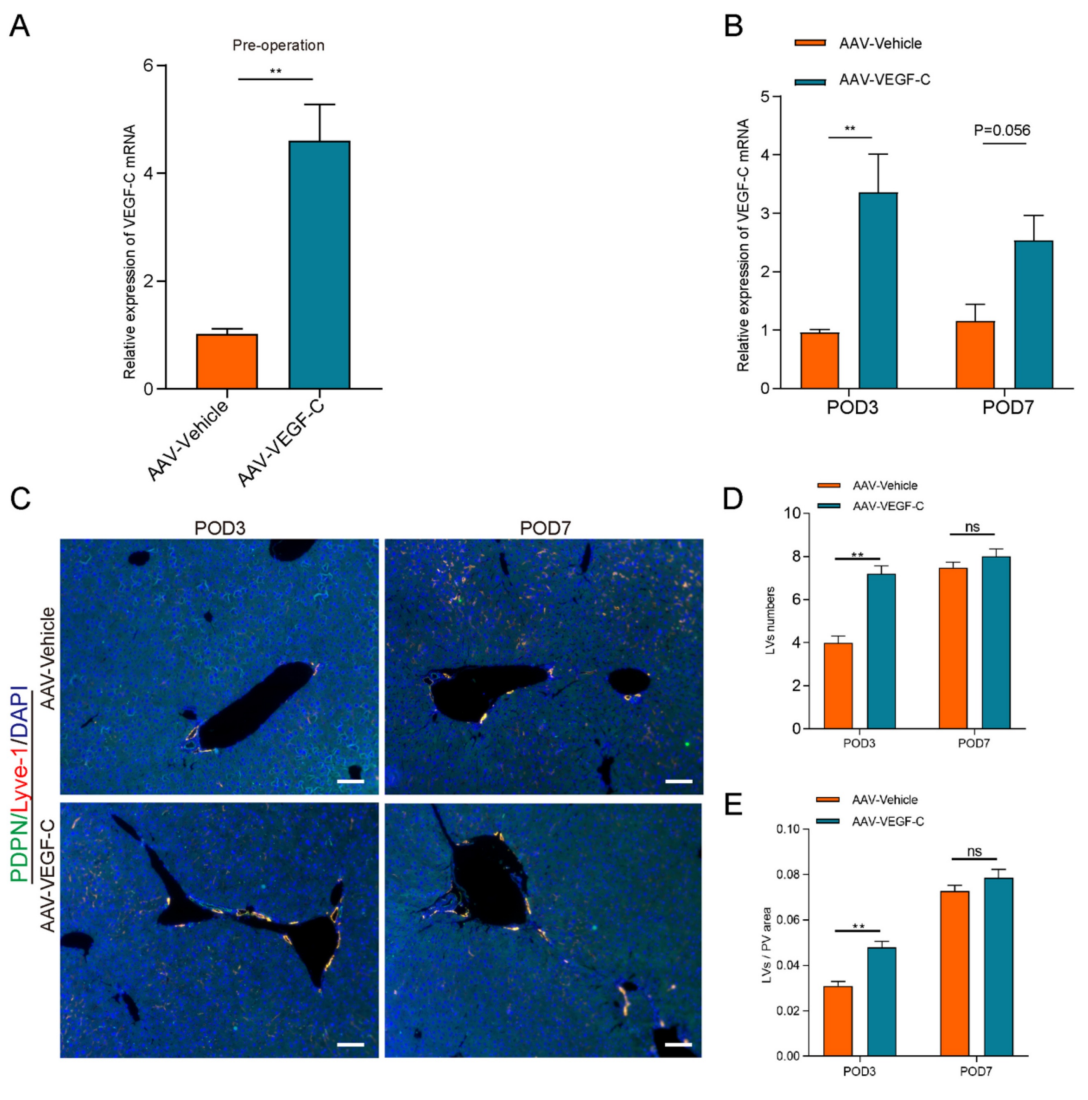
** Administration of AAV-VEGF-C stimulates lymphangiogenesis in the liver.** A: The expression level of VEGF-C in the liver was detected via qRT-PCR on pre-operation (n=5 per group, 5 biological replicates from 5 individual animals). B: The expression of VEGF-C in the liver was detected via qRT-PCR on POD3 and POD7 groups (n=5 per group, 5 biological replicates from 5 individual animals). C: Representatives of immunofluorescence co-staining of LYVE-1(red) and PDPN(green) in the liver among POD3, POD7(n = 5 per group, 5 biological replicates from 5 individual animals; original magnification 100X, scale bar: 100μm). D: Statistical analysis of LVs numbers in immunofluorescence staining. E: Statistical analysis of LVs area in immunofluorescence staining. These results were obtained from at least three independent experiments. Values are presented as mean ± SEM. ∗∗*p* < 0.01, ∗*p* < 0.05, ns > 0.05.

Our current study showed that the number and area of LVs were decreased on POD3 compared to sham group in the process of liver regeneration. However, the mRNA level of VEGF-C was increased on POD3. Increased VEGF-C expression in response to vessel damage represents a compensatory mechanism for restoring lymphatic function. Therefore, we examined whether the administration of AAV-VEGF-C contributes to intrahepatic lymphangiogenesis. As shown in the Figure 3A, AAV-VEGF-C group had higher mRNA expression level of AAV-VEGF-C compared with the AAV-Vehicle group on pre-operation. These data indicated that adeno-associated virus-mediated overexpression of VEGF-C was successfully achieved. In addition, the mRNA expression level of VEGF-C also increased significantly in AAV-VEGF-C group on POD3 compared to the AAV-Vehicle group without significant difference on POD7(Figure 3B). Consistent with the mRNA expression levels of VEGF-C, both the number and area of lymphatic vessels (LVs) were significantly increased in the AAV-VEGF-C group on POD3 compared to the AAV-Vehicle group, with no significant difference observed on POD7 (Figure 3C-E). These data collectively indicated that the administration of VEGF-C stimulated intrahepatic lymphangiogenesis.

### Lymphangiogenesis contributed to accelerate liver regeneration and repair

**Figure 4:**
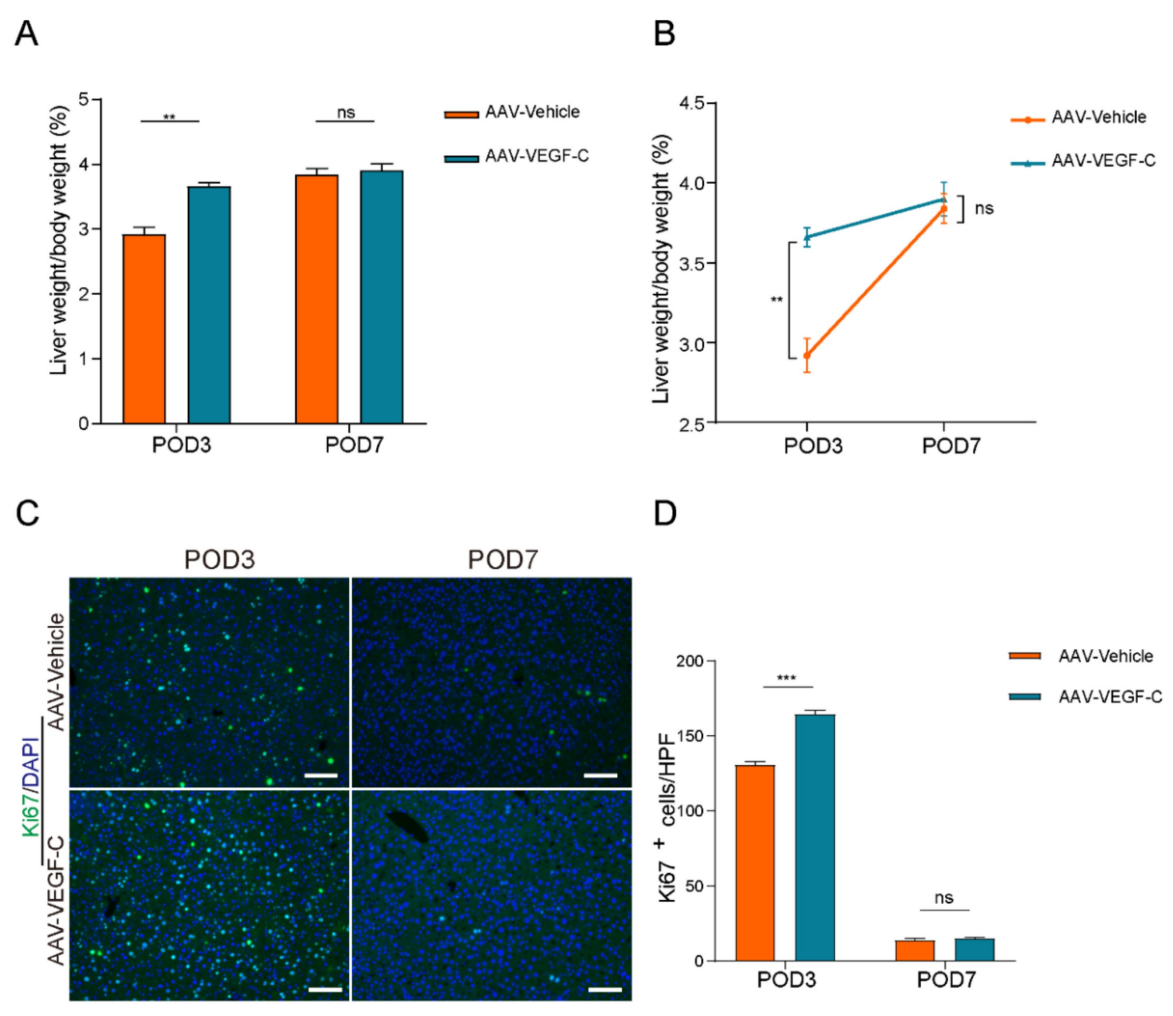
** Lymphangiogenesis contributed to accelerate liver regeneration.** A and B: The liver weight to body weight percentage was detected on POD3 and POD7 (n=5 per group, 5 biological replicates from 5 individual animals). C: Representatives of immunofluorescence staining of Ki67 in the liver among POD3 and POD7(n = 5 per group, 5 biological replicates from 5 individual animals; original magnification 100X, scale bar: 100μm). D: Statistical analysis of positive Ki67 cells in immunofluorescence staining. These results were obtained from at least three independent experiments. Values are presented as mean ± SEM. ∗∗*p* < 0.01, ∗*p* < 0.05, ns > 0.05.

**Figure 5:**
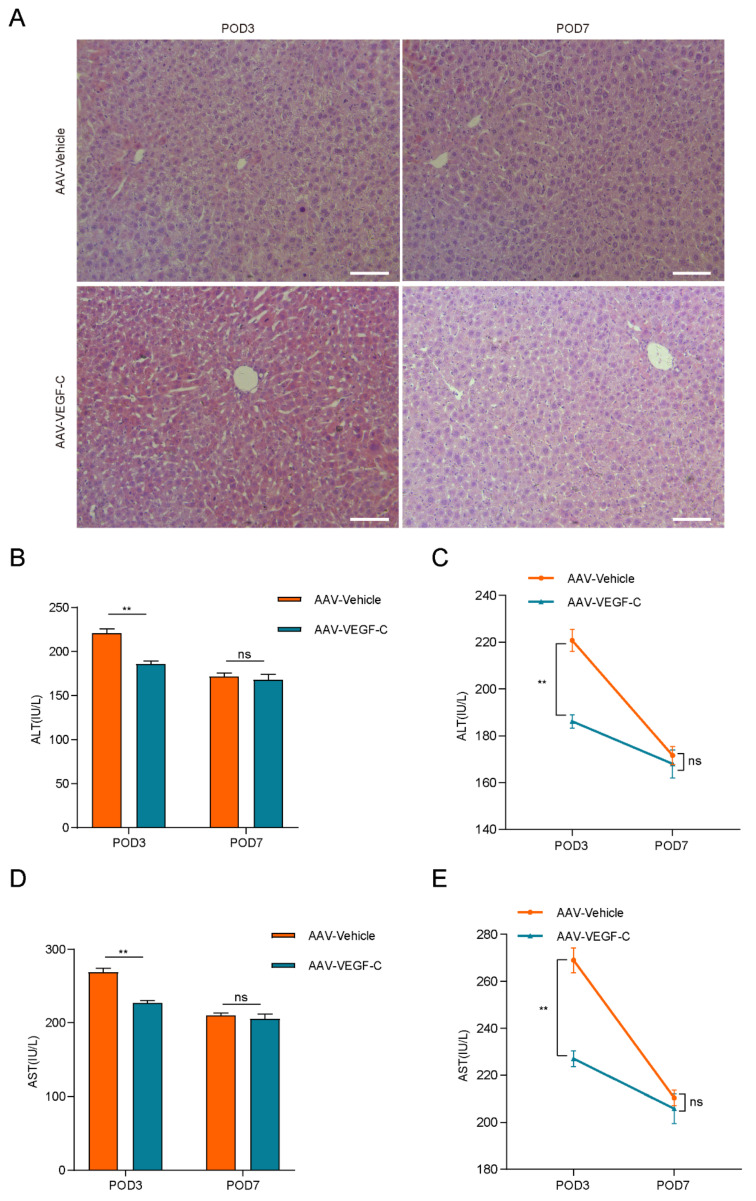
** Lymphangiogenesis contributed to the promotion of liver repair.** A: HE staining was performed to assess live injury and repair in mice (n = 5 per group, 5 biological replicates from 5 individual animals; original magnification 100X, scale bar: 100μm). B and C: Serum ALT level of the AAV-Vehicle and AAV-VEGF-C groups were detected (n = 5 per group, 5 biological replicates from 5 individual animals). D and E: Serum AST level of the AAV-Vehicle and AAV-VEGF-C groups were detected (n = 5 per group, 5 biological replicates from 5 individual animals). These results were obtained from at least three independent experiments. Values are presented as mean ± SEM. ∗∗*p* < 0.01, ∗*p* < 0.05, ns > 0.05.

As show in Figure 4A-B, the percentage of liver weight to body weight increased significantly on POD3 in the AAV-VEGF-C group compared to the AAV-Vehicle group, nearly reaching the level observed on POD7. There was no significant difference on POD7, which indicated that promoting intrahepatic lymphangiogenesis advanced the liver regeneration peak from POD7 to POD3. Furthermore, intrahepatic Ki67-positive cells of AAV-VEGF-C group also significantly increased on POD3 compared to the AAV-Vehicle group without significant difference on POD7 (Figure 4C-D). In addition, although there was no significant difference between AAV-Vehicle group and AAV-VEGF-C group in HE staining (Figure 5A), but the level of ALT and AST presented significant decrease in AAV-VEGF-C group on POD3 without significant difference on POD7(Figure 5B-E). All these findings indicated that promoting lymphangiogenesis contributed to accelerate liver regeneration and repair following 70% PHx.

### Lymphangiogenesis promoted liver regeneration by activating of IL-6/STAT3 pathway

**Figure 6:**
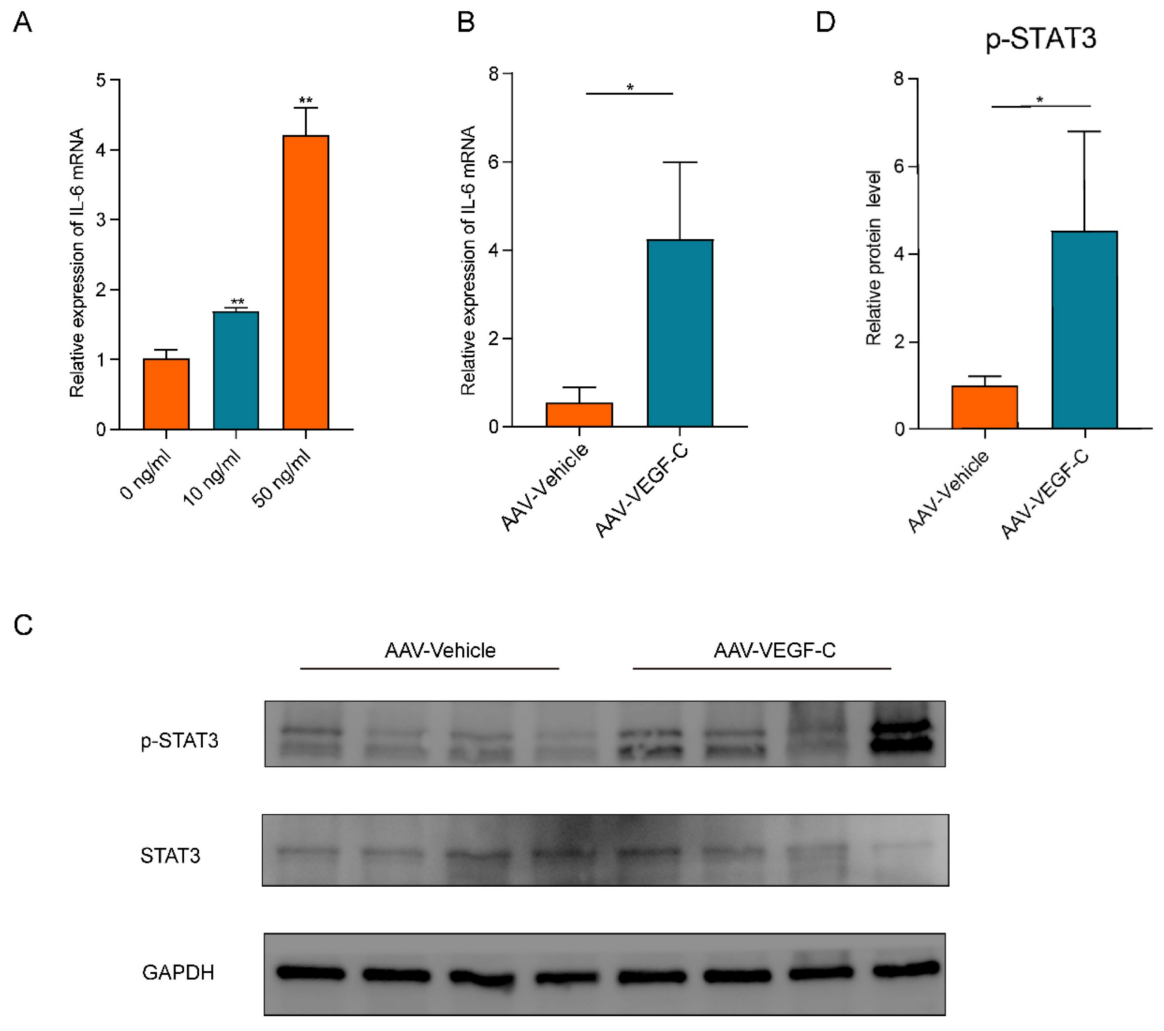
** Lymphangiogenesis promoted promoted liver regeneration by activating of IL-6/STAT3 pathway. A:** IL-6 mRNA expression levels in LyECs stimulated with VEGF-C *in vitro***.** B: The expression of IL-6 in the liver was detected via qRT-PCR on POD3 (n=5 per group, 5 biological replicates from 5 individual animals). C-D: The relative protein level of pSTAT3 in the liver were detected via western blotting on POD3 (n=5 per group, 5 biological replicates from 5 individual animals). These results were obtained from at least three independent experiments. Values are presented as mean ± SEM. ∗∗*p* < 0.01, ∗*p* < 0.05, ns > 0.05.

Given the extensive involvement of IL-6 in lymphangiogenesis^20^, ^34^, we measured IL-6 mRNA expression levels in human LyECs stimulated with VEGF-C *in vitro* (Figure 6A). Our data shown that VEGF-C stimulated human LyECs to secrete IL-6 in a concentration-dependent manner. As shown in Figure 6B, the mRNA levels of IL-6 were also significantly increased in liver tissues from the AAV-VEGF-C group on POD3 compared to the AAV-Vehicle group. Additionally, IL-6 is a crucial cytokine that activates STAT3 in hepatocytes, playing a key role in hepatocyte proliferation following 70% PHx^35^. As shown in Figure 6C-D, the protein levels of p-STAT3 in liver tissue were also significantly elevated in the AAV-VEGF-C group on POD3 compared to the AAV-Vehicle group. These data collectively suggested that lymphangiogenesis may accelerate liver regeneration and repair by activating the IL-6/STAT3 pathway.

### Blocking IL-6 reversed lymphangiogenesis-accelerated liver regeneration

**Figure 7:**
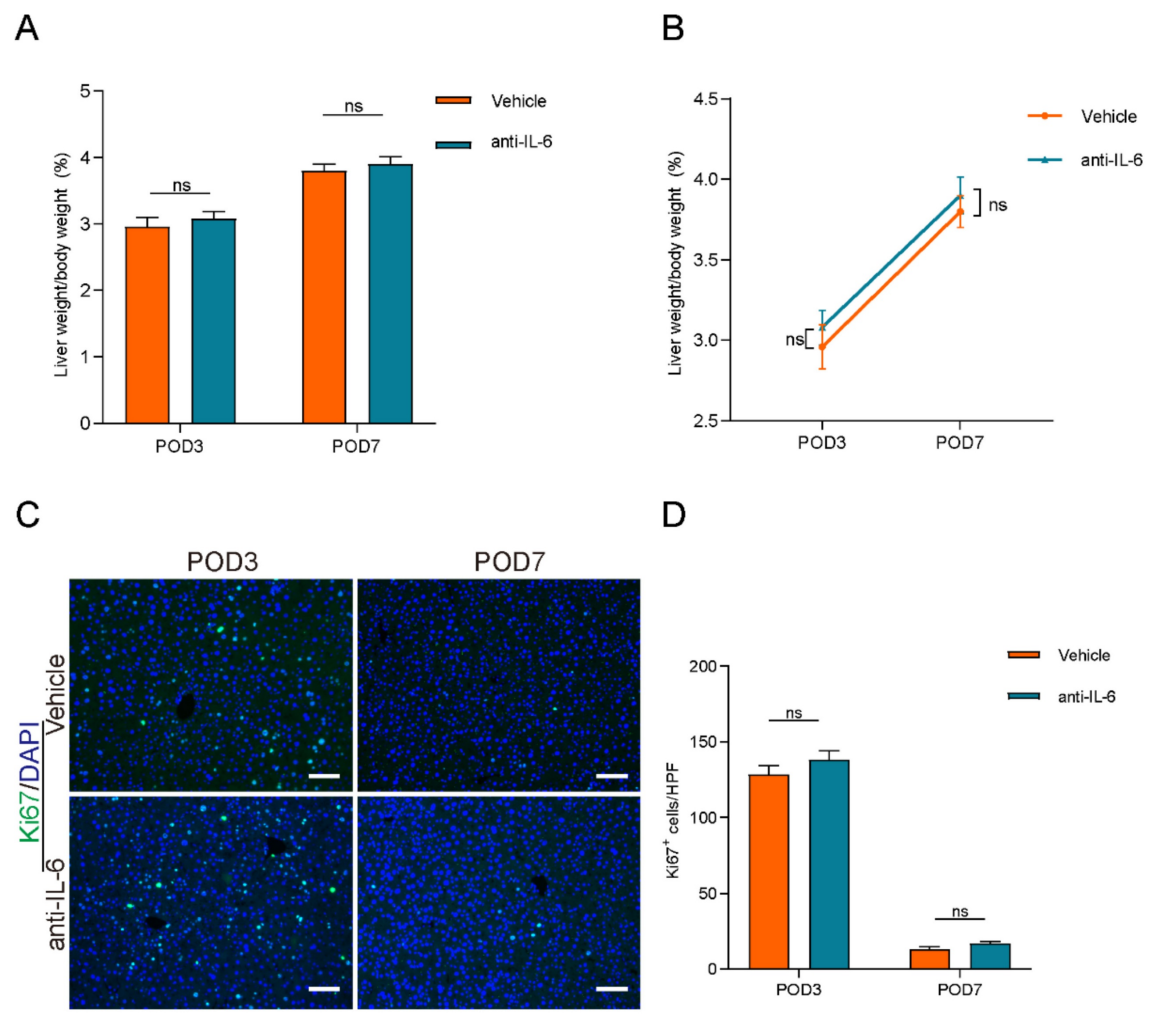
** Blocking IL-6 reversed lymphangiogenesis-accelerated liver regeneration.** A and B: The liver weight to body weight percentage was detected on POD3 and POD7 between Vehicle group and anti-IL-6 group (n=5 per group, 5 biological replicates from 5 individual animals). C: Representatives of immunofluorescence staining of Ki67 in the liver among POD3 and POD7(n = 5 per group, 5 biological replicates from 5 individual animals; original magnification 100X, scale bar: 100μm). D: Statistical analysis of positive Ki67 cells in immunofluorescence staining. These results were obtained from at least three independent experiments. Values are presented as mean ± SEM. ∗∗*p* < 0.01, ∗*p* < 0.05, ns > 0.05.

To investigate the role of the IL-6/STAT3 pathway in lymphangiogenesis-accelerated liver regeneration, we administered anti-IL-6 protein to block IL-6 signaling. The efficacy of this treatment was confirmed by a reduction in STAT3 phosphorylation levels, as shown in Figure S3. However, as depicted in Figures 7A and 7B, there were no significant differences in the percentage of liver weight to body weight between the anti-IL-6 and vehicle groups. Additionally, intrahepatic Ki67-positive cell counts did not differ significantly between these groups (Figures 7C and 7D). These findings suggested that blocking IL-6 signaling reversed the acceleration of liver regeneration induced by lymphangiogenesis.

## Discussion

The lymphatic vascular system is believed to play a role in the regeneration and repair of various tissues. In heart, promoting lymphangiogenesis enhanced cardiac regeneration and repair following injury^36^. Cardiac lymphangiogenesis was also required for exercise-induced physiological cardiac growth by VEGFR3 activation^37^. As for bone, lymphangiogenesis induced by genotoxic stress stimulates bone regeneration by secreting CXCL12^23^. Although lymphangiogenesis have been observed in liver ischemia reperfusion injury^10^, its significance for post-hepatectomy liver regeneration remains unclear. Here, we demonstrated that intrahepatic lymphangiogenesis changes dynamically in the process of liver regeneration following 70% PHx.

Our data indicated that lymphangiogenesis played a significant role in the process of liver regeneration and repair following 70% PHx. Our single-cell RNA-sequencing analysis revealed that both the proportion and function of LyECs in liver tissues undergo dynamic changes following 70% partial hepatectomy. Our study also indicated that liver regeneration was accompanied by a reduction in the number of LVs on POD3, and that lymphangiogenesis accelerated liver regeneration and repair. Furthermore, our study demonstrated that lymphangiogenesis accelerates liver regeneration through the activation of the IL-6/STAT3 signaling pathway. These findings enhance our understanding of the roles of intrahepatic LVs in liver regeneration. Moreover, despite the growing insight into this intricately regulated and complex process, enhancing liver regeneration remains a challenging endeavor. Delayed liver regeneration, particularly following hepatectomy, continues to be associated with significant morbidity and mortality^38^. Thus, advancing liver regenerative capacity holds significant potential for improving patient outcomes.

Our results revealed that both the number and area of intrahepatic LVs significantly decreased on POD3 following 70% partial hepatectomy, but returned to pre-operative levels by POD7. Lymphangiogenesis is a complicated process with various cytokines and growth factors and signaling pathways. There are several potential explanations for these results. The liver has been considered as a crucial organ for lymph production and regulation due nearly 25%-50% of lymph passing through the thoracic duct originates from the liver^39^. When liver mass loss suddenly following 70% PHx, the amount of lymph also decreased. Portal venous blood flow of per unit liver volume also inevitably decreased and leads to hepatic lymph fluid decreased^40^. These lead to a significant decrease in liver lymphatic flow, resulting in a notable decrease in lymphatic pressure^41^. The body may employ feedback mechanisms to regulate lymphatic fluid and lymphatic pressure, potentially inhibiting lymphangiogenesis to maintain balance^42^. So intrahepatic LVs numbers decreased around the portal tract on POD3. In addition, liver injury resulted in the production of large amounts of pro-lymphangiogenic factors, such as IL-1β,IL-6,VEGF-C, and the liver quickly responded to lymphatics formation and expand to draining them^43^. Among of them, VEGF-C is a ligand that binds to its receptor of VEGFR3 and activates downstream signaling pathways that stimulates LyECs proliferation and migration. VEGFR3 is expressed not only on LyECs but also on other types of cells such as macrophages^10^. What's more, macrophages also produce VEGF-C in an autocrine manner driving lymphangiogenesis^44^. When the lymphangiogenic factors were more than anti-lymphangiogenic factors, intrahepatic LYs numbers were recovered around the portal tract on POD7.

Our results showed that the mRNA expression level of VEGF-C was inversely related to the number of LVs in POD3. Due to the damage to intrahepatic LVs following 70% PHx, the increased expression of VEGF-C may serve as a compensatory response to this LVs damage^33^, ^45^. When LVs were damaged or compromised—whether due to inflammation, mechanical injury, infection, or pathological conditions like cancer or chronic inflammation—the body often upregulates VEGF-C to promote the repair and regeneration of the lymphatic network. There are several reasons why this reaction might work. Firstly, damaged or inflamed tissues release signals that induce VEGF-C production by local cells, such as fibroblasts, macrophages, or epithelial cells, to encourage lymphatic repair^46^. Second, VEGF-C's role in lymphangiogenesis is especially critical in attempts to regenerate damaged vessels^47^. High VEGF-C levels aim to stimulate LyECs to proliferate and migrate, ideally replacing or restoring lost or dysfunctional vessels. This is a protective mechanism designed to restore lymphatic function. Third, persistent damage or high levels of VEGF-C signaling may lead to a self-amplifying cycle where VEGF-C expression is continuously elevated, particularly if lymphatic regeneration is slow or hindered by other factors^48^, ^49^. This feedback mechanism ensures that VEGF-C remains available to support any possible lymphatic regeneration when the vessel integrity is compromised. However, in cases where LVs repair is unsuccessful or inhibited by ongoing tissue stress, the elevated VEGF-C may not rapidly prevent LVs loss or regression. It is consistent with our results that the number and area of LVs decreased on POD3 and restored on POD7.

To further examine the relationship between lymphangiogenesis and regeneration, mice received AAV-VEGF-C prior to 70% PHx. These animals showed increases in periportal LV number and area, a higher LBW ratio at POD3 (with no difference by POD7), a higher Ki-67 index at POD3, and lower ALT at POD3. Collectively, these data are consistent with VEGF-C overexpression exerting its main impact during the early phases of liver regeneration following PHx, at least in part by enhancing lymphangiogenesis. Enhanced lymphangiogenesis may facilitate the clearance of inflammatory mediators and the establishment of a favourable reparative microenvironment in the early phase^50^. Increased lymphatic vessel formation at POD3 may also be associated with augmented IL-6 production, which has been implicated in promoting early liver repair. However, by POD7, other regenerative signals and compensatory mechanisms—such as hepatocyte proliferation and the contribution of alternative angiogenic factors—likely become dominant, diminishing the relative impact of VEGF-C overexpression. This temporal shift suggests that VEGF-C is particularly important during the early “priming” phase of liver regeneration, whereas its relative contribution decreases as the repair process progresses. Importantly, systemic AAV-VEGF-C delivery can exert pleiotropic effects beyond lymphatic endothelium^51^, ^52^. Even liver-tropic vectors (e.g., AAV8 under hepatocyte-directed promoters) may show extrahepatic transduction and off-target physiological effects, and the durability of expression can reflect a mixture of episomal maintenance and occasional vector integration—features that complicate strict tissue-specific attribution. Accordingly, we interpret the AAV-VEGF-C findings as associative and avoid lymphatic-endothelium-specific causal claims. Overall, within these bounds, VEGF-C-linked augmentation of lymphatic indices appears to align with accelerated early regenerative kinetics and reduced injury markers during the initial postoperative phase.

All the results indicated that lymphangiogenesis had an additional contributed roles in the process of liver regeneration following 70% PHx. LVs exhibit a variety of immunoregulatory functions by expressing a wide range of chemokines and receptors^53^. The drainage function of LVs plays a crucial role in the resolution of inflammation. In addition, our data indicated that lymphangiogenesis activated IL-6/STAT3 pathway in the process of liver regeneration following PHx. IL-6 is one of the major inflammatory interleukins that has been linked to liver regeneration^19^, ^54^. Blocking IL-6 reversed lymphangiogenesis-accelerated liver regeneration. We provided evidence that STAT3 activation contributes, associated to IL-6 secretion by LyECs. It is likely that LyECs secreted IL-6 in certain circumstances, such as liver regeneration circumstances. Van de Velde *et al.* also reported that tumor exposed LyECs, but not normal LyECs, produced huge amount of IL-6 which exerted mitogenic effect on tumor cells in the primary tumor^20^.

In our current study, we primarily focused on elucidating the role of VEGF-C-induced lymphangiogenesis in promoting liver regeneration. While we did not specifically investigate the direct effects of VEGF-C on liver sinusoidal endothelial cells (LSECs) or analyze Wnt signaling pathways within these cells, we acknowledge that LSECs—which are known to express VEGFR3—may also be influenced by VEGF-C and could contribute to the regenerative process^55^. Lymphangiogenesis can coincide with the accumulation of reparative macrophages, and VEGF-C/VEGFR-3 signaling has been implicated in shaping macrophage recruitment and programs^10^. To improve specificity with the available markers, periportal lymphatic vessels were defined by dual positivity for PDPN and Lyve-1 together with wall-forming tubular morphology and an erythrocyte-poor lumen, explicitly excluding sinusoid-like, non-wall-forming Lyve-1^+^ networks that typify hepatic sinusoids^56^. In our scRNA-seq dataset, LyECs expressed canonical lymphatic transcripts (Flt4/Vegfr-3, Prox1), providing orthogonal transcript-level support for lymphatic identity despite the known limitation that Lyve-1 is not liver-specific for lymphatics^25^. Nevertheless, time-resolved, protein-level Vegfr-3 IHC/IF was not performed and remains an important limitation. In our experiments, LyECs exhibited significant increases in IL-6 secretion and STAT3 activation, both of which are known to promote lymphangiogenesis and tumor progression^20^. This aligned with previous findings showing that IL-6 induces VEGF-C expression in lymphatic endothelial cells via the Src-FAK-STAT3 signaling pathway, further enhancing lymphangiogenesis^57^. Additionally, IL-6 has been shown to promote tumor growth, invasion, and lymphangiogenesis in gastric cancer through the JAK-STAT3-VEGF-C axis^58^. Therefore, our data support an association between VEGF-C-induced lymphangiogenesis and activation of the IL-6/STAT3 pathway during liver regeneration, although additional mechanistic studies.

Taken together, while our study provides novel insights into the involvement of lymphangiogenesis in liver regeneration, it also has several limitations. First, although POD3 and POD7 are critical time points following 70% PHx, our assessment of intrahepatic lymphangiogenesis was restricted to these intervals, and the optimal window during which lymphangiogenesis most strongly influences regeneration remains unknown. Second, because VEGF-C was administered systemically and PHx priming is inherently multifactorial, residual confounding by other processes (e.g., macrophage dynamics, angiogenesis, LSEC responses) cannot be excluded. Third, the potential contribution of LSECs in the VEGF-C overexpression model was not specifically addressed, and future studies will be required to dissect the relative roles of LSECs versus LyECs. Finally, although we preliminarily explored the roles and mechanisms of lymphangiogenesis in liver regeneration using a murine 70% PHx model, further validation in human samples is warranted.

In conclusion, intrahepatic LVs were decreased on POD3 and recovered on POD7 in the process of liver regeneration following 70% PHx. Lymphangiogenesis promoted by AAV-VEGF-C significantly accelerated liver regeneration and repair following 70% PHx, which might be associated with regulating IL-6/STAT3 pathway. Targeting lymphangiogenesis might be a potential strategy for advancing liver regeneration.

## Supplementary Material

Supplementary figures and table.

## Abbreviations

AAV: Adeno-associated virus
AAV-VEGF-C: Adeno-associated virus overexpressing vascular endothelial growth factor-C
ALT: Alanine aminotransferase
anti-IL-6: Anti-IL-6 antibody
AST: Aspartate aminotransferase
Cdh5: Cadherin 5
Gja1: Gap Junction Alpha-1
GEO: Gene Expression Omnibus
IL-6: Interleukin-6
Jam2: Junctional Adhesion Molecule 2
LVs: Lymphatic vessels
LyECs: Lymphatic endothelial cells
PHx: Partial hepatectomy
PHLF: Post-hepatectomy liver failure
POD: Postoperative day
SFSS: Small-for-size syndrome
STAT3: Signal transducer and activator of transcription 3
Tjp1: Tight Junction Protein 1
VEGF-C: Vascular endothelial growth factor-C
