# Supplementary material for: Intrahepatic Lymphangiogenesis Is Associated with Early Post-Hepatectomy Liver Regeneration, in Part via IL-6/STAT3 Signaling
Source: Int J Med Sci. 2026 Jan 14;23(2):646–60. doi: 10.7150/ijms.106849 (PMC12825147; doi:10.7150/ijms.106849)
Supplement: Supplementary file 1 — Supplementary figures and table. [file ijmsv23p0646s1.pdf]

Supplementary materials: Figure S1, S2 and S3.

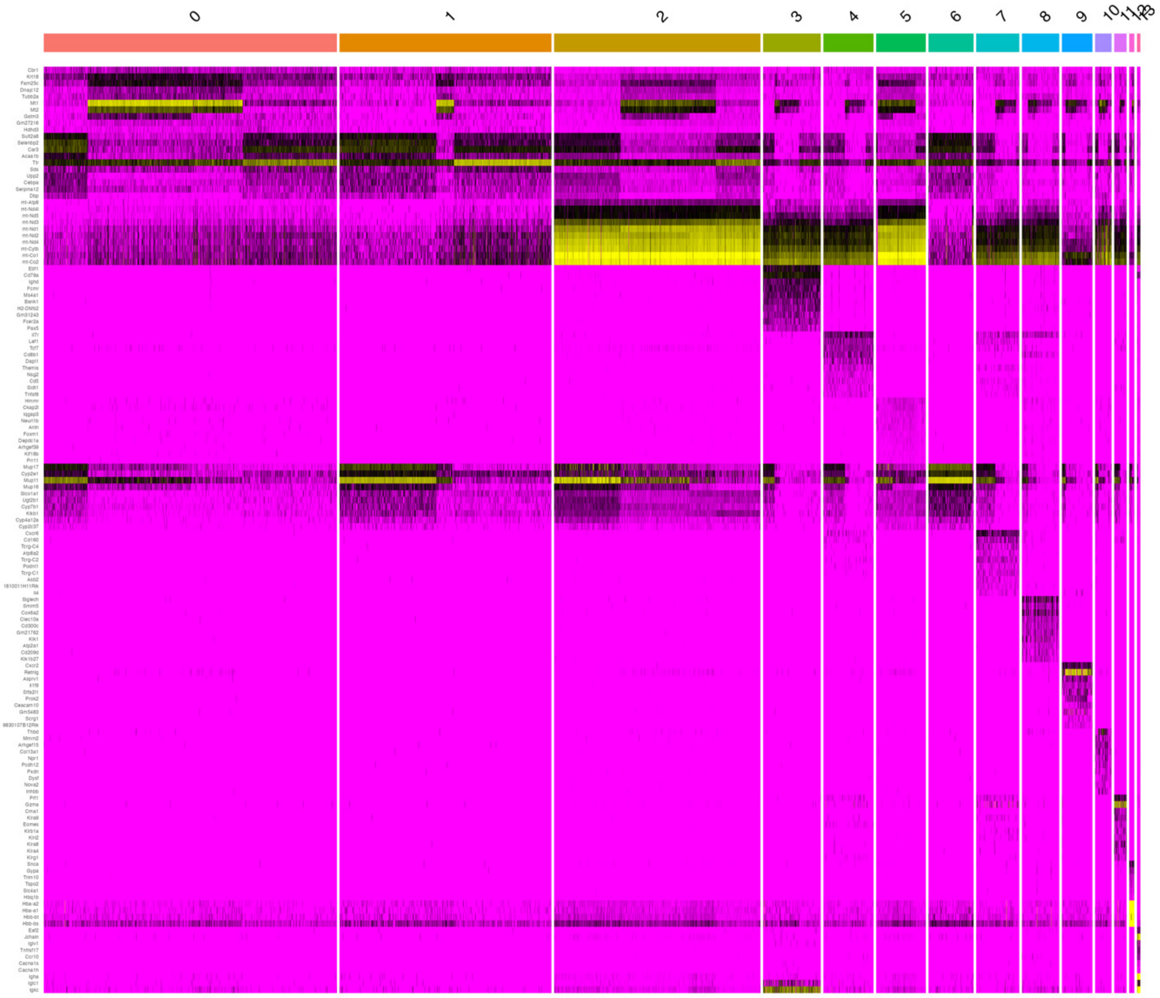

Figure S1. The TOP10 genes of these subpopulations.

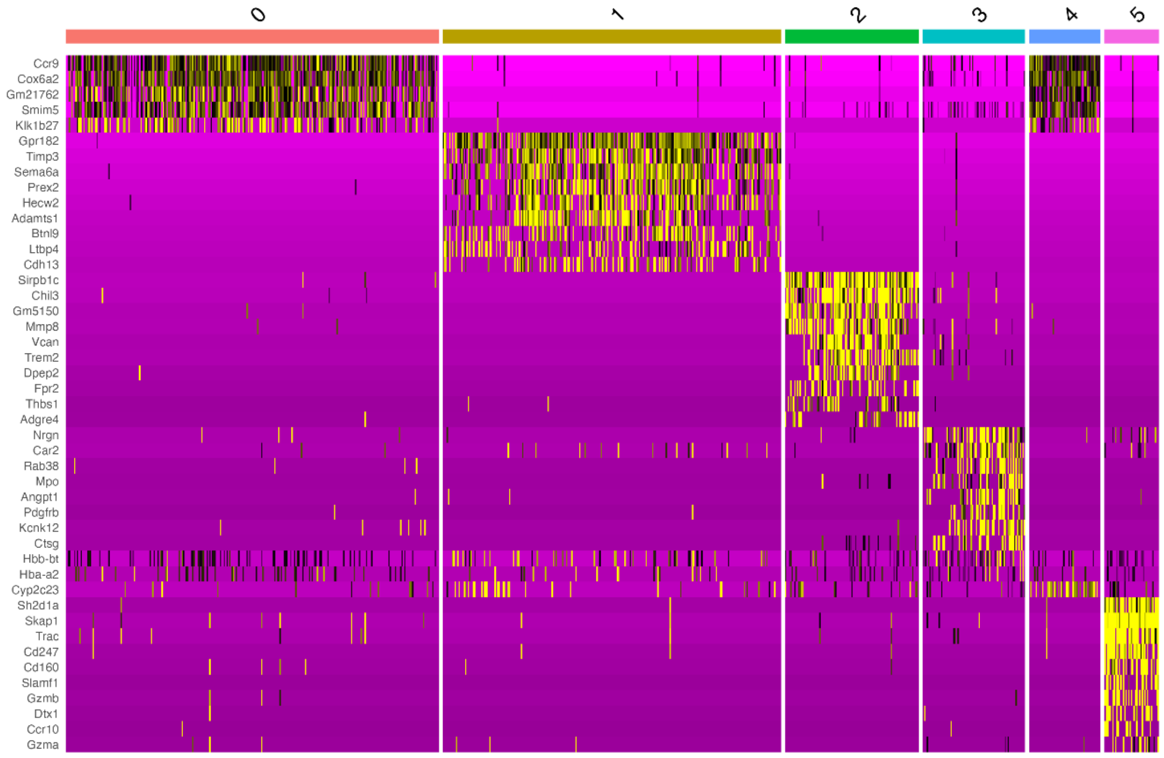

Figure S2. The TOP10 genes of endothelial cells.

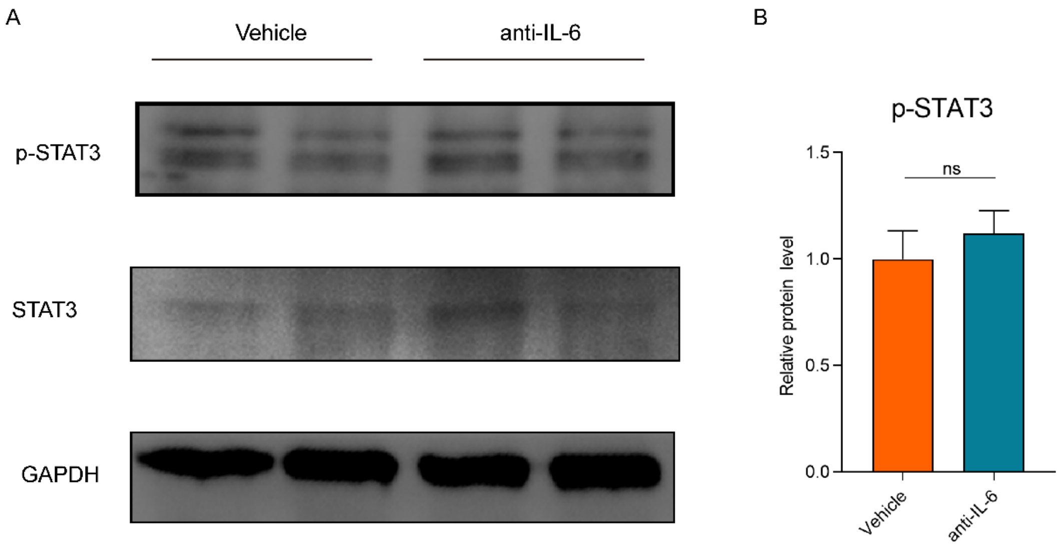

Figure S3. The anti-IL-6 treatment efficiency. A-B: The relative protein level of pSTAT3 in the liver were detected via western blotting on Vehicle and anti-IL-6 groups (n=5 per group, 5 biological replicates from 5 individual animals). These results were obtained from at least three independent experiments. Values are presented as mean ± SEM. $**p < 0.01$, $*p < 0.05$, ns > 0.05.

Table S1 The sequence of the primers for qRT-PCR

Gene	Species	Forward primer	Reverse primer
IL-6	*Mus musculus*	5'CTGCAAGAGACTTCCATCCAG3'	5'AGTGGTATAGACAGGTCTGTTGG3'
VEGF-C	*Mus musculus*	5'GTGAGGTGTGTATAGATGTGGGG3'	5'GTCTTGCTGAGGTAACCTGTG3'
GAPDH	*Mus musculus*	5'CACTGAGCAAGAGAGGCCCTAT3'	5'GCAGCGAAATTTATTGATGGTATT3'
IL-6	*Homo sapiens*	5'ACTCACCTCTTCAGAACGAATTG3'	5'CCATCTTTGGAAGGTTCAGGTTG3'
